# Ameliorating a Complex Urban Ecosystem Through Instrumental Use of Softscape Buffers: Proposal for a Green Infrastructure Network in the Metropolitan Area of Naples

**DOI:** 10.3389/fpls.2019.00410

**Published:** 2019-04-03

**Authors:** Emanuela Coppola, Youssef Rouphael, Stefania De Pascale, Francesco Domenico Moccia, Chiara Cirillo

**Affiliations:** ^1^Laboratory of Territorial Planning, Department of Architecture, University of Naples Federico II, Naples, Italy; ^2^Department of Agricultural Sciences, University of Naples Federico II, Portici, Italy

**Keywords:** biodiversity conservation, ecological network analysis, fragmentation habitat, land consumption, urbanization, zoning

## Abstract

Green Infrastructure (GI) definition, deriving from the United States green infrastructure for hydro-geological rebalancing through imitating the nature stormwater management, was consolidated in Europe by GI Planning Guide. Nowadays GI can be considered a valid and meaningful approach for ameliorating urban complex ecosystems, and could also be considered as mitigation action of land consumption, according to the guidelines on the soil sealing of the European Commission (2012). The metropolitan area of Naples located in south Italy is characterized by an unauthorized and chaotic urban development. The land-use map reported an average of 30% of urbanization in the metropolitan area, rising up to 50–60% and as high as 98% in the north core area of the city. This high level of urbanization is directly related to the habitat fragmentation. The National Biodiversity Conservation Strategy defines several challenges and targets to counteract the biodiversity loss in Italy, identifying urban areas as places exposed to the greatest pressures on ecosystems. Therefore, the integration of different policies limiting habitat fragmentation, heat island effect and natural soil hydro-geological degradation into spatial planning, especially through green corridors and ecosystem enhancement in urban areas is an urgent need for the society. Spatial planning has to be renewed in metropolitan areas, where threats and weaknesses to biodiversity conservation are stronger than in any other place, according to the Law n. 56/2014, (Gazzetta Ufficiale della Repubblica Italiana, 2014) committing metropolitan cities to the enactment of General Territorial Plan. In the current paper, we aim at designing an ecological network for the metropolitan area of Naples one of the biggest city of southern Italy. The analyses include the adopted methodological procedure, i.e., ecological network analysis and design, and the introductory elements of a spatial analysis on a pilot ecological network of several patches. Finally, the paper illustrates the network analysis conceived as a monitoring system and also in future perspective, as a planning support system.

## Introduction

In 1995, the European Ministers of the Environment in the framework of the new-born Pan European biological and landscape diversity strategy (PEBLDS), aiming to strengthen the environmental politics and maintenance of the biodiversity, established the Pan European Ecological Network (PEEN). The PEEN road map was developed in the Council of Europe (1996) aiming “*to design and develop an ecological network among European states. It will consist of core areas, corridors and buffer zones. Restoration areas will be identified where they are considered necessary. Moreover, PEEN aims to conserve the full range of ecosystems, habitats, species and landscapes of European importance and to counteract the main causes for decline by creating the right spatial and environmental conditions*.”

While “*Core areas and great Insulae*” are defined as areas of high biodiversity value which act as hubs for the Green Infrastructure (GI; such as Natura 2000 areas), therefore corresponding with natural or semi-natural habitats, the “buffer zone” correspond to the edge zone to the core areas that assume a protective function toward for the core areas. Additionally, among structural elements of the network besides the well known “corridors,” defined by Forman (1995) as *“strips of a particular type differing from the adjacent land on both sides*,” there are some isolated areas of potential ecological connection, namely “stepping zones,” that are constituted by small environmental patches representing, due to their position and/or composition, essential elements of the landscape for the maintenance of the connectivity for the vegetal/animal species (ISPRA, 2019). On the other hand, the PEEN include also the peri-urban permeable areas at elevated fragmentation, called “restoration areas,” that are of crucial importance in the territories of the metropolitan city of Naples, where the processes of fragmentation have reached elevated critical levels (Mininni et al., 2001). Through interventions of environmental restoration, these areas can become of fundamental importance for the ecological transformation of the metropolitan city of Naples (Mininni et al., 2002). In the past two decades, urban ecosystems are becoming increasingly important as contributors to both the problems and potential solutions to counteract these important environmental issues. In particular, the loss of agricultural and natural landscapes will place greater pressure on urban green spaces to provide, rather than simply production and cultural resources that were available from rural areas in the past, relevant ecosystem functions, representing the main prerequisite for the subsistence of ecosystem services (Kandziora et al., 2013; Pettorelli et al., 2017). Moreover, among the categories of ecosystem services, besides production and cultural services, an increased importance has been assumed by regulation and support services (Pataki et al., 2011). Urban green spaces, for example, will have an important role in conserving biodiversity, protecting water resources, limiting soil sealing, improving microclimate, sequestering carbon, as well as supplying a portion of fresh food consumed by urban dwellers. At the same time, these spaces must continue to meet the traditional cultural needs of nearby residents by encouraging recreational activities, embodying the aesthetic preferences of the community, educating people about nature and preserving historic landscape features. These various functions, which provide the “ecosystem services” that benefit humans either directly or indirectly, will need to be considered simultaneously and to be balanced to meet the needs and preferences of local residents as well as society as a whole (Lovell Taylor and Taylor, 2013).

Taking this background into consideration, the aim of the present study was to propose an operative methodology for the construction of a green network in the particular metropolitan area of Naples (Mininni et al., 2001), as a central point for the ecological transformation of the city (Moccia, 2010). The intervention methodology suggested, aiming to increase the development of areas with restored environmental services within the city, may represent a suitable and generalizable tool. Therefore, obtained results can play an important role in ameliorating complex urban ecosystem in several European metropolitan areas.

## Materials and Methods

### The Study Area

**FIGURE 1:**
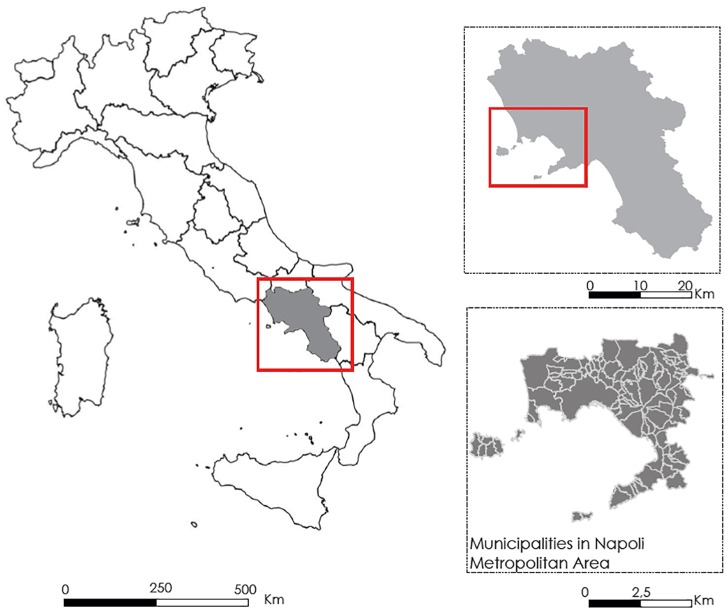
The study area of the Metropolitan City of Naples (Italy).

The Metropolitan City of Naples has been instituted in 2014 by the Gazzetta Ufficiale della Repubblica Italiana (2014). Naples is one of the biggest metropolitan district in Italy, being third in terms of number of inhabitants and first for housing density (Moccia and Sgobbo, 2017). It includes the municipality of Naples and ninety-one towns. Among the natural elements that characterize its landscape, there are the Vesuvius in the middle part, the northern volcanic area of Campi Flegrei and also the Mounts Lattari in the peninsula of Sorrento (Figure 1).

### Operative Methodology for the Construction of the Metropolitan Green Network

The reconstruction of the natural sites, prior to the urbanization, is based on geo-morphology and hydro-graphic system analysis (Coppola, 2016). Following the main environmental planning theories (Mc Harg, 1989; France, 2002) the adopted methodology for a Green Infrastructure (GI) project (Moccia, 2013; Moccia and Coppola, 2013; Coppola, 2016) originates from an historical and geographical interpretation of the area under investigation, being considered as analytical step needed to implement planning principles to the “*design with nature”* by the United States Environmental Protection Agency (EPA) able to identify GI practices to improve the water-ecological balance of the territory and based on a network of public spaces and permeable areas – gray or green areas – that we can reach in pedestrian mode.

According to the indications provided by the Regional Territorial Plan (Regione Campania, 2008), the construction of the metropolitan ecological network is also based on agricultural policies and great infrastructures as pivotal items for the construction of the network. In particular, agricultural areas whilst they maintain their economic productivity, may be rearranged handing the environmental sustainability mandates, and can constitute the minimal network of connections between the area’s most preserved from the environmental point of view and with a high level of biopermeability (Regione Campania, 2008).

On the other hand, great linear infrastructures, such as highways, railway, drainage channels and large electric lines, when designed or restructured following appropriate criteria, may represent also a set of complementary elements to the supporting structure for the construction of the regional ecological network, and also contribute to the connection of the most important natural areas (Regione Campania, 2008). In the construction process of such ecological networks, the role of municipal-scale planning become pivotal, because it is a determinant action, through the design of land use plan, giving completeness to the landscape planning through the active involvement of local actors in the main steps of identification and recognition of landscape values (Risser et al., 2007). Therefore, the main analyses provided by this methodology were a general landscape analysis that included an evaluation of biodiversity and soil fertility status. Furthermore, a detailed investigation on the fragmentation elements was also performed.

### Landscape Analysis

**FIGURE 2:**
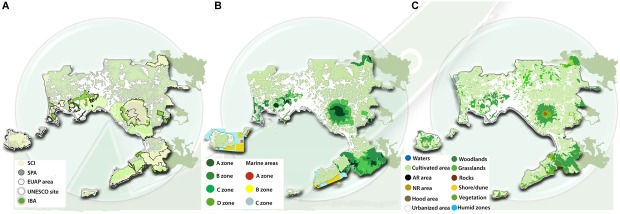
Landscape analyses of the study area of the Metropolitan City of Naples: **(A)** distribution of Special Protection Areas (SPA) and Sites of Community Importance (SCI) in the framework of Natura 2000 standards, areas included in the Italian Ministry of Environment and Water Management official list of protected areas (EUAP), UNESCO sites and Important Bird Areas (IBA); **(B)** distribution of parks and reserves; **(C)** land cover use (AR, artificial recolonization; NR, natural recolonization).

The first step has been the construction of maps of the environmental system of the studied area. Indeed, in the Metropolitan City of Naples the natural elements constitute a sort of wide, and integrated in the urbanized tissue, environmental reserve. The natural heritage of Naples is particularly rich and includes several protected areas (Figure 2) representing also a cultural, touristic and economic resource. The Community Interest Sites (SIC) defined by the Council Directive 2006/105/Ec (2006), together with the Special Protection Areas (SPA), UNESCO sites and Important Bird Areas (IBA), were reported on a map (Figure 2A). Once the protected areas have been localized, they have been also classified under the point of view of the in force legislation (Italian Ministry of Environment and Water Management, 2010) by mapping and classifying the main categories (Table 1 and Figure 2B). One national park for the Vesuvio volcano, four regional parks (Campi Flegrei, Mounts Lattari, Hydrographic Basin of Sarno and Partenio) and the metropolitan park of Naples hills are located within the borders of the metropolitan city, from the northern to the southern boards of the city (Figure 2B), representing the potential outline of a GI network (core areas). Furthermore, even though in this area the urbanization process happened in a confused and spread way generating both an expansion of the metropolitan areas and an urban sprawl (Mazzeo, 2009), the analysis of the land cover highlighted that the agricultural use of the soil still represents on average a 39% of the metropolitan area (Figure 2C). In particular, the agricultural connotation become stronger in some areas such as the eastern part of Vesuvio and the peninsula of Sorrento and in the western part the Campi Flegrei, where more than half of the territory still maintain an agricultural function. In general, more than 25% of the metropolitan area is represented by forests, tree orchards and vineyards that are mainly grown in the north-east of Vesuvio, whereas a lower percentage of the cultivated area is allocated to vegetable crops (10%) as well as greenhouses and tunnels (1.7%) located in the northern area of Acerra and in the plain of Pompei (Figure 2C).

**Table 1:** Classification and description of terrestrial and marine protected areas included within the borders of the Metropolitan City of Naples (Italy), following the in force legislation.

	Protection level	Description	Allowed interventions	Not allowed interventions
Terrestrial areas				
	A	High environmental, naturalistic value sites. Habitat development and conservation in its integrity. Naturalistic, scientific, education and cultural use.	Conservation: - restoration and management aiming to soil protection and hydraulic risk mitigation - ecosystemic quality protection	Any kind of intervention hindering conservation of the site.


	B	High naturalistic value sites. Diffuse presence of woodlands and traditional agriculture, Ecosystemic function increase and biodiversity conservation. Connection areas with A zones. Naturalistic, scientific, education and cultural use Allowed also sports, recreational and touristic activities. Traditional agricultural systems at high historical and cultural value (eg., terraces) and conservative productive forestry.	Conservation priorities: - forestry, traditional agriculture and naturalistic walk - management aiming to soil protection and hydraulic risk mitigation - ecosystemic quality protection.	Any kind of intervention affecting soil conformation and hydrographic system, but its original restoration.
	C	Naturalistic, environmental and landscape values strictly connected to specific cultural, agricultural and rural models. Management, restoration and redevelopment of agriculture and forestry. Biodiversity conservation. Gradual replacement of residential functions with agricultural functions.	Rural and touristic infrastructure redevelopment	Any kind of intervention affecting soil conformation and hydrographic system, but riverbed and cave rehabilitation and restoration.
	D	Deeply modified sites as result of anthropic activities. Activities and services supporting the valorisation and fruition of A, B, and C areas. Social and economical development of local communities.	Management, redevelopment and transformation of hydrographic system and urbanization to minimize hydrological and volcanic risks.	Any kind of intervention aiming to residential use.
Marine areas	A	Integral reserve.	Scientific research	Any activity disturbing or damaging the marine natural environment
	B	General reserve.	Very low environmental impact activities	
	C	Partial reserve, acting as buffer zone between A, B and external areas.		


**FIGURE 3:**
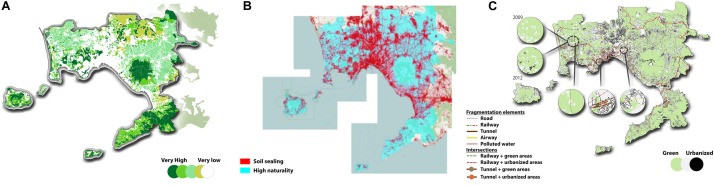
Biodiversity **(A)**, soil sealing **(B)** and fragmentation **(C)** analyses of the study area of the Metropolitan City of Naples.

The precise recognition and design of these components is a key step in the project of an ecological network, especially in an urban area where, despite a significant abundance of plant vegetation and biodiversity (Figure 3A), the soil-sealing is very high (Figure 3B). According to the analysis of “structure environmental functional historian” of the PTC (previously known as PTCP) of the Metropolitan City of Naples (2016), the analysis of the fertility of the soils has been implemented. The Plan of Territorial Coordination (PTC) of the metropolitan area of Naples aims to promote the increase of the Ecological Network through the recognition of new green parks and sites in which to foresee interventions of re-naturalization. In the general design, the protected areas shape themselves as excellent knots of an Ecological Network, connecting the principal natural systems of Campi Flegrei, Vesuvius – Somma Mountain and Sorrento Peninsula.

The analysis of the plant biodiversity, started from Corine Land Cover data (2012), and aimed to the assessment of the biodiversity in landscape mosaics of the agricultural landscapes, by using, as landscape metrics, portions of natural, semi-natural, and intensive land uses (Walz, 2011). This approach confirmed that, among the agricultural uses of the soil of the studied area, horticultural destinations and forestry still prevail. Furthermore, with the aim to classify the environmental quality through the definition of a vegetal biodiversity level of the agro-systems, it has been defined a qualitative grading, based on the land cover in the Metropolitan City of Naples (2016). Specifically, five levels of plant biodiversity were defined from very low to very high according to the main incidence of agrosystem types in the towns and municipalities of the studied area (Table 2 and Figure 3A), that allowed to highlight the possibility to develop some strategical ecological corridors able to connect the existing main core areas within the borders of the metropolitan city of Naples.

**Table 2:** Classification of levels of biodiversity based on main land cover and their distributions within the Metropolitan City of Naples (Italy).

Level	Land use	Areas	Municipalities
Very low	greenhouses/tunnels	Northern area, Vesuvian coastal area, South-western area	Acerra, Afragola, Caivano, Ercolano, Torre del Greco, Poggiomarino, Sant’Antonio Abate, Santa Maria la Carità and Pompei
Low	open field vegetable crops, industrial crops and spring-summer cereals	North-western retro coastal area, North-eastern area	Giugliano in Campania, Pozzuoli, Acerra, Marigliano, Nola, San Vitaliano, Pomigliano
Intermediate	fall-winter cereals, alfalfa pasture, fruit tree orchards	North-western area, Eastern Vesuvian area	Giugliano in Campania, Qualiano, Villaricca, Quarto, Marano e Sant’Antimo, Sant’Anastasia, Poggiomarino e Casamarciano
High	olive and citrus orchards, vineyards, natural and artificial recolonization areas, post-fire natural areas	Coastal area of Sorrento Peninsula, Vesuvian area, North-eastern area, Campi Flegrei area and Islands	Sorrento Terzigno Nola Pozzuoli, Capri, Ischia and Procida
Very high	chestnuts, forests, permanent grasslands and pastures, Mediterranean brush, shores and dunal areas, coastal and internal humid areas	Northern area Vesuvius area Southern area	from Ischia Island to Hills of Naples all around the volcano from Capri Island to the Sorrento peninsula


### Analysis of the Fragmentation

The environmental and landscape fragmentation is a process of anthropic origin consisting in the subdivision of one environmental patch type (i.e., grass, wood etc.) in smaller and more isolated fragments. In the present study the ecological fragmentation intensity has been evaluated by using Soil Monitor Web-Gis (2016) tool developed at the CRISP Research Center (University of Naples Federico II and the National Researches Center) with the collaboration of ISPRA, Geosolutions and National Institute of Urbanism (INU) on 2016. This innovative tool, that interacts with the data-banks ISPRA and GIS informative bases, allows to appraise, to monitor and to quantify the soil-sealing and the ecological fragmentation of the municipalities of the Italian metropolitan cities. The available functions in web-GIS are “urban fragmentation” and “rural fragmentation” and may be calculated on 200 m sized pixels. The mathematical function used is the following,

$$F_{P}\;=\;\frac{\sum_{k\;=\;1}^{n}V_{k}}{n\;-\;1}$$

where *Fp* is the fragmentation of the pixel at the center of kernel and *V_k_* is the value of the pixel (except *Vp*) within the k kernel of n pixels.

This analysis showed a high level of fragmentation in the metropolitan area (Figure 3B) due to the wide spread of impermeability within the area, even though several areas of greater naturalness co-exist. The main elements of fragmentation are represented by roads and railways that break and intercept natural homogeneous patches by returning smaller ecological island that negatively affect the landscape structure and perception (Figure 3C).

## Results and Discussion

The effects of urban ecosystems on biodiversity have been widely investigated by several authors (Wackernagel and Rees, 1996; Beckline and Yujun, 2014), since the impact of a city on adjacent ecosystems may raise significantly (Douglas, 1983). On the contrary, the application of biodiversity study to urban ecosystems analyses and consequent planning strategies represents a more recent approach (Savard et al., 2000).

### Construction of the Ecological Corridors

Regional and local governments can implement several actions aiming to increase biodiversity in urban ecosystems that includes planning, design and management at different landscape scale (Botequilha Leitão et al., 2006). Once the regional policies have already designed parks and protected areas and identified the main green corridors linking the city to natural areas, it become hierarchically crucial to plan the extension of green corridors within the city. Therefore, Municipal government is in charge to shape, structure and size up corridors to optimize plant vegetation and animal abundance and biodiversity inside the urban areas (Savard et al., 2000).

**FIGURE 4:**
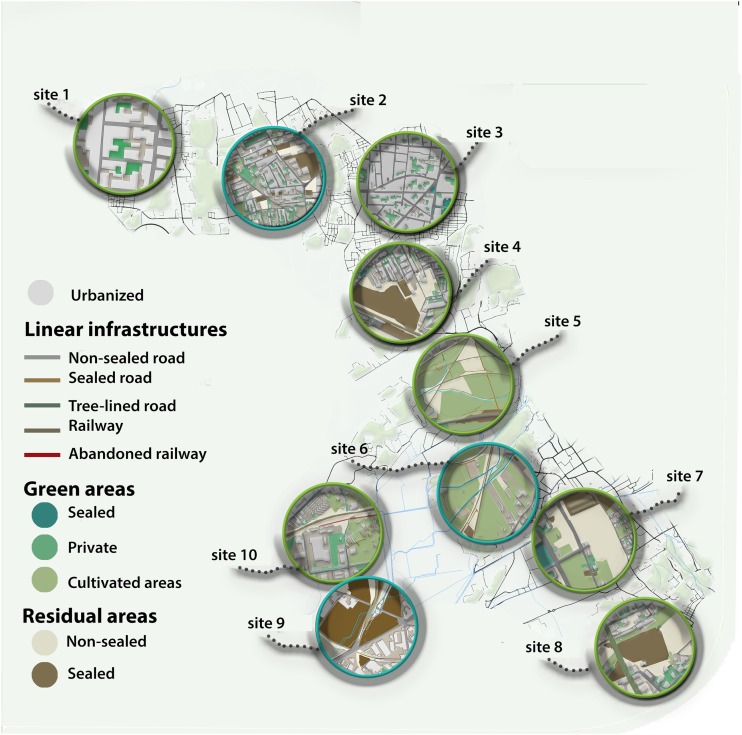
Sites (from 1 to 10) along the potential ecological corridor connecting the two core areas of the Hills of Naples and the Vesuvius, selected for the application of different types of interventions.

All the analyses carried out led to define the possible strategies of implementation of a functional green corridor in the highly urbanized metropolitan area of the city of Naples. Planning and designing ecological corridors needed to start from the punctual definition of the main core areas. They have been identified in the great national, regional and metropolitan parks, already existing in the city area. Among these, the National Park of Vesuvius that acts as an important nodal point, results to be also a spatially central point, from which connections may depart to other Parks, such as Metropolitan Park of the Hills in Naples. Among the potential corridors that have been traced, only one have been selected and well deepened in the present work (Figure 4), since it resulted to be the most interesting for lower degree of fragmentation and for the peculiar “building up” methodology applied in the final section of zoning strategies and solutions.

### Reversing Fragmentation Elements in Ecological Connections

In strongly urbanized contexts, the infrastructural network frequently determines an interruption of the ecological continuity (Berruti et al., 2014). In this project of green network in urban circle, the urban infrastructures may become the skeleton of the ecological network. All the buffer areas have been individualized and categorized in:

- Agricultural areas, divided in two categories: the arable land and olive-groves, vineyards, fruit orchards and chestnut. These areas already have by itself an elevated biodiversity and they must be protected.
- Public areas (permeable), uniforms in two categories: the first one, constituted by all those areas to public use that don’t have a specifies destination, usually abandoned and often use as dump. The second one, constituted by the areas of road pertinence such as bands of respect, grass and trees.
- Private permeable areas, of dimensions visibly more redoubts, are generally constituted by private gardens or commons gardens.
- Impervious areas as zones that can contribute to a best result in the ecological network realization if suitable changes were brought. These areas include: the transition zones (asphalted abandoned areas where the nature returned, are used often as deposit of various kind refusals); the parking areas (or parts of them), often public or of pertinence of industries, therefore generally of elevated greatness and completely asphalted.

At the same time the elements of fragmentation were also cataloged, as follows:

- the skyways and the underpasses of highways and railways. The elevated section produce a different level of fragmentation in comparison to the roads.
- the canalized rivers and tombed canals: the artificial canalizations, both underground and superficial, constitute a barrier to the roads. These structures have cemented banks and can became as ecological traps.

The objective was the maintenance and the recovery of the riverside environmental conditions and therefore maintaining the landscape values, taking in account also the historical and original distribution of blue areas within the cities (Iojă et al., 2018). According to the principles of Landscape Planning, GI “*use soil and vegetation for infiltration, evapotranspiration and/or recycling of rainwater*.” When used as components of rainwater management systems, GI, such as green roofs, permeable floors, rain gardens, and green trenches, can provide a variety of environmental benefits (Bortolini and Zanin, 2018). In addition, the GI allows the storm water sedimentation and infiltration, these technologies can reduce air pollutants and energy demand, mitigate the effect of urban heat island and retain carbon monoxide, but – at the same time-they offer communities aesthetic benefits and green spaces (Galagoda et al., 2018).

### The Project of the Strategic Ecological Corridor

**FIGURE 5:**
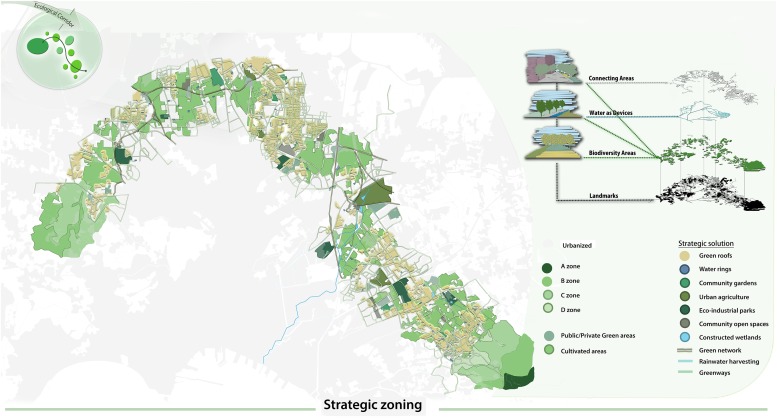
Strategic zoning solution application to the potential ecological corridor connecting the two core areas of the Hills of Naples and the Vesuvius.

In this strategy of construction of the metropolitan ecological network a step-by-step layering approach has been adopted (Figure 5), where the protected areas may represent the knots of the Ecological Network, while the hydrographic network may allow to define the ecological corridors characterized by a high index of biodiversity. Landscape structures supporting the connectivity of species, biological communities and ecological processes are recognized as key elements of conservation in human altered environments (Walz, 2011). Nevertheless, the potential connections from the national park of the Vesuvius, the main core area, to the other protected and/or natural parks located within (Hills of Naples, Campi flegrei and Mounts Lattari) or at the borders (Mount Partenio) of the Metropolitan City of Naples are frequently crossed by the existing linear infrastructures that constitute the barriers to strengthen this connections, since they interrupt the environmental continuity in the area.

In particular, the implemented corridor connecting the national park of the Vesuvius with the metropolitan hills Park in Naples may have a significant positive impact on one of the most fragmented part of territory (Figure 5).

In highly urbanized contexts, the definition of GI includes a rather large type of green space but also gray areas. Indeed, GI is the physical environment within and between our cities, towns and villages. It is a network of multi-functional open spaces, including formal parks, gardens, woodlands, green corridors, waterways, street trees and open countryside. It includes all environmental resources, as well as a GI approach and contributes toward sustainable resource management (Davies et al., 2007).

### The Zoning Solution

A taxonomy of project-solutions, useful to resolve some recurrent problems of the (ri)creation of the green network come out of former studies resorting to two different but complementary parameters: ecological and urbanistic parameters. The ten recommended interventions were differentiated on the base of the state of fact: urbanized zones, road network, waters, residual and agricultural areas (Figure 4, Table 3).

**Table 3:** Qualitative grading and matrix of possible interventions in the ten different selected sites distributed along the potential strategical corridor connecting two of the main core areas (Hill of Naples and Vesuvius area) within the Metropolitan City of Naples.

			Selected sites									
			
			site 1	site 2	site 3	site 4	site 5	site 6	site 7	site 8	site 9	site 10
Ecological parameters	Biodiversity		0^∗^	1	0	1	3	3	1	0	0	2
	Streams	Polluted	0	0	0	0	4	4	0	0	4	0
		Non-polluted	0	0	0	0	3	0	0	0	3	3
	Green areas	Public	0	0	1	1	0	0	1	2	0	0
		Private	2	4	4	3	0	0	2	2	0	1
Urbanistic parameters	Roads	Sealed	4	4	4	4	0	0	1	2	3	3
		Non-sealed	0	0	0	0	4	4	0	0	0	2
	Residual areas	Sealed	0	2	0	3	1	1	3	3	4	0
		Non-sealed	0	1	0	3	3	3	4	3	3	2
Cultivated areas			0	1	0	1	4	4	3	2	0	3
Intervention type^∗∗^			GR	WR	CG	UAP	EIP	COS	CW	GN	RH	GW


*^∗^0, very low; 1, low; 2; intermediate; 3, high; 4, very high. ^∗∗^GR, green rooftops; WR, water rings; CG, community gardens; UAP, urban agricultural parks; EIP, eco-industrial parks; COS, community open spaces; CW, constructed wetlands; GN, green networks; RH, rainwater harvesting; GW, greenways*.

The main types of interventions were defined as follows:

• Green Rooftops (GR) were based on the possibility of install green roofs to increase biodiversity (Orsini et al., 2014). While there are many examples of United States green cities – that have already chosen to apply GI systems in great commercial centers during the last quarter of a century, forcing private owners to achieve a vegetation standard of 30% of the surface built through green roofs and walls and permeable floors (Coppola, 2016). In Mediterranean countries such as Italy and Greece there is still today a serious limitation in their application (Tsantopoulos et al., 2018).
• Water Rings (WR) are drainage systems provided in residual residential spaces that collect rainwater slowing down the flow rate and returning it purified through micro-processes of phytodepuration (France, 2002). Each system is designed to capture rainwater to be collected into retention ponds and driven to laminate.
• Community Gardens (CG) represent residual permeable areas in densely populated neighbor hoods. The municipal plans can use this function enhancing these actions in this way. Community Garden have a dual objective: to improve the quality of life of citizens and to produce benefits for the entire community. The city of Bologna has identified four types of free areas as places for implementing urban gardens: flowerbeds along streets and squares, balconies and roofs, abandoned buildings and abandoned neighborhoods (Gasperi et al., 2016). Coherently, in all urban spaces, areas that present a lack of identity can be converted into urban agricultural areas and, more specifically, in urban horticulture as a way to strengthen resilience and sustainability (Gasperi et al., 2016).
• The Urban Agricultural Parks (UAP) represent great permeable areas where urban agriculture becomes a fundamental element to redevelop the landscape but also to improve the quality of the landscape and social life (Mancebo, 2018) Thus, we can propose addresses for the care of the territory through the agricultural culture practiced by different subjects and for different purposes.
• Eco-industrial Parks (EIP) are programmed, designed and managed on the basis of the principles of ecology and the circular economy as a key to building a new society based on the themes of resilience, efficient use of resources and environmental protection, directed toward a sustainable development (Ward Thompson, 2002).
• The Community Open Spaces (COS) are urban common areas used as urban standards, including recreational areas and playgrounds (La Rosa and Privitera, 2013), very often abandoned and degraded. These areas represent central redevelopment areas and nodes of the urban green network where their connection determines its effectiveness.
• The Costructed Wetlands (CW) are based on phytoremediation systems that are fundamental for reconstructing the environmental ecosystem, especially in those areas where the river ecosystem are compromised (Moccia and Berruti, 2018). Furthermore, some linear projects such as green network, rainwater harvesting and greenways were also included (Figure 4).
• Green network (GN), as green addition along the roads, have the effect of reducing the heat island, increasing the biodiversity and linking the different residual areas and natural patches (Malcevschi, 2010).
• Rainwater Harvesting (RH) are a rainwater collection system that includes components of various phases (rainwater transport through pipes or drains, filtration and storage in tanks for reuse or recharge). The reuse of rainwater collected can be reused to irrigate the agricultural areas, for domestic hygiene and could be used by industries (Moccia and Berruti, 2018).
• Greenways (GW) are the recovery of abandoned tracks to rebuild/enhance the ecosystem and to be a link with the residual and potential areas (Coppola and Vanella, 2016).

These interventions are embedded in local plans through zoning regulations. The advantage of incorporating the green infrastructure corridor and its connecting ecological funtions in the urban planning document is to have a general and complete framework also if implementasion may occur in a segmented way with many projects carried out in different periods.

## Conclusion

The planning of interventions from the metropolitan scale to the municipal area is the central node to implement these strategies. Urban plans and many projects based on the principles of urban sustainable infrastructures are growing in different regions around the globe. The first European approaches were the Green Belts, an instrument of urban policies to control urban expansion and to protect landscapes. They are green rings designed to protect the consolidated city which, providing an appropriate mix of agriculture, forestation and recreational activities, can effectively counteract the urbanization. In the United Kingdom, Green Belts covered the 13% of the British territory, Spain created the Barcelona *Anellaverda* and the Territorial Planning in the metropolitan area of Lisbon created one more Green Belt. In Italy, there are only three examples of green belts in the cities of Turin, Ferrara and Mirandola (Modena).

To make effective the realization of such green areas, it is necessary that the municipal plans allow similar interventions in the different zones of the plan through the urban planning regulations. More restrictive regulations have to be applied to the historical centers, where only community garden interventions and some types of public spaces (squares, parking areas, and multifunctional spaces), since linear projects (green network, rainwater harvesting, and greenways) may result too difficult to realize. On the contrary, regulations might be more concessive in the consolidated areas, where it is also possible to intervene on the buildings with rooftop gardens and water rings, but also community open spaces and linear projects are possible. In recent construction areas and in specialized areas (i.e., commercial and industrial) most of the interventions are possible.

In Italy, the use of sustainable urban infrastructures are still very limited to individual local initiatives and are not included in a extended network that is essential for the achievement of green objectives as well as European funds. The metropolitan plans of the All London Green Grid in London and the GI plan in New York are dedicated plans that design only sustainable urban infrastructures to increase urban resilience. Inspired by these international experiences, the conference “The Nature of Italy” (13–14 December 2013) recommended to prepare – in Italy too – a Metropolitan Plan for the Restoration of Ecological Continuity (PRCE) and the methodology proposal in this paper goes toward this goal.

Finally, in our cities, the green infrastructures are multifunctional environmental components, that should be recognized and explicitly included in metropolitan and urban planning, and a highest priority of funding from EU commission should be given to their construction.

## Author Contributions

CC, FDM, and EC conceived and planned the work. CC, SD, and EC carried out the data processing and analysis. EC, CC, and YR drafted the initial manuscript, which was critically revised by all authors.

## Conflict of Interest Statement

The authors declare that the research was conducted in the absence of any commercial or financial relationships that could be construed as a potential conflict of interest.
